# Epigenetic changes found in uterine decidual and placental tissues can also be found in the breast cancer microenvironment of the same unique patient: description and potential interpretations

**DOI:** 10.18632/oncotarget.23488

**Published:** 2017-12-19

**Authors:** Miguel H. Bronchud, Francesc Tresserra, Bernat Serra Zantop

**Affiliations:** ^1^ Institut Bellmunt Oncologia, Hospital Universitari Dexeus, Grupo Quiron Salud, Barcelona, 08028 Spain; ^2^ Servicio de Anatomía Patológica y Citología, Hospital Universitari Dexeus, Grupo Quiron Salud, Barcelona, 08028 Spain; ^3^ Servicio de Ginecología, Obstetricia y Reproducción, Hospital Universitari Dexeus, Grupo Quiron Salud, Barcelona, 08028 Spain

**Keywords:** materno-fetal tolerance, cancer microenvironment, placental microenvironment, immune vigilance, carcinogenesis

## Abstract

Microenvironmental properties are thought to be responsible for feto-maternal tolerance. Speculatively, ectopic expression of placental gene programs might also be related to cancer cells’ ability to escape from immune vigilance mechanisms during carcinogenesis and cancer progression. Recently, we published the first human genomic evidence of similar immune related gene expression profiles in both placenta (placenta and decidual tissue) and cancer (both primary and metastatic) in the same patient with lymph-node positive breast carcinoma during pregnancy. Here we report the first epigenomic analysis of these tissue samples and describe their main findings, with respect to immune related genes regulation (over or under expressed) in cancer cells with regards placental tissues. We confirm significant similarities, and hierarchical clustering (both unsupervised and supervised), in CpG island methylation patterns between decidual/placental and cancer microenvironments, which cannot be easily explained by simple models or unique pathways. Several different cell types are probably involved in these complex immune regulation mechanisms. Cancers may somehow “hijack” gene programs evolved over millions of years to allow for feto-maternal tolerance in placental mammals in order to escape from immune vigilance and spread locally or to distant sites.

## INTRODUCTION

Complex and multiple immune related mechanisms allow the uterus and the placenta to mount powerful responses to infection (bacterial, protozoal and viral), but at the same time to tolerate fetus’ paternal allo-antigens, both inside the mother’s womb and in the blood circulation of both mother and fetus [1]. Five days after fertilization, the human zygote develops into a structure consisting of 2 primary cell lines: the inner cell mass (or embryoblast) and the trophoblast [2]. Trophoblast cells constitute the outer layer of the blastocyst, and rapidly proliferate and invade the maternal endometrial decidua around day 7. A monolayer of cytotrophoblast cells surrounds the embryonic disc as the embryo completely embeds beneath the uterine decidua. By day 9, cytotrophoblast cells have differentiated into 2 distinct cell types: the syncytiotrophoblast and the extravillous trophoblast (EVT). The multinucleated syncytiotrophoblast cells form the external layer and are terminally differentiated. These cells are involved in fetomaternal nutrient exchanges and endocrine functions (such as β-human chorionic gonadotropic). In contrast, EVT cells have a proliferative and invasive phenotype, migrating through the syncytiotrophoblast into the uterine wall to anchor the placenta beginning around day 14 after implantation [3]. These EVT cells display a phenotype strikingly similar to cancer cells, with capacity for proliferation, migration and establishment of blood supply, making them a compelling model for oncologic comparison [4].

During pregnancy, the endometrium becomes a specialized tissue (named decidua), with a very special microenvironment characterized by placental tissue invasion and a high proportion of leukocytes (40–70%). These leucocytes have unique regulatory functions to allow both for fetus’ immune defense against microbial pathogens and feto-maternal tolerance. The major immunological regulating cellular populations are: macrophages, dendritic cells (also antigen presenting), regulatory T cells (Treg), and uterine decidual natural killer cells (dNK cells). All these play important roles in establishing tolerance and maintaining the homeostatic environment that is crucial for normal fetal development. Endothelial cells and fibroblasts may also be indirectly involved. Human fetal placenta itself, mainly through trophoblast cells and cytokines, is able to induce homeostatic M2 macrophages and Tregs [5].

The different immune related genes similarly over or under expressed in cancer and placental microenvironments — that we recently described by applying Nanostring genomics [6] — are unlikely to be due to a “single regulatory cell or pathway”, but are more likely to be the combined result of gene expression changes of cytokines, receptors, transcription factors, and other immune regulatory genes (of the 750 panel tested), in several if not all of these four main stromal immune cellular types. At present we are unable to propose any single immune regulatory pathway to explain these gene expression patterns which apparently confer “placental/decidual properties” to cancer tissues (primary breast cancer and metastatic LN) of our single clinical-molecular case report. In this paper, we analyze the same tissues (normal breast, malignant breast, normal axillary lymph node, metastatic axillary lymph node, uterine decidua and placenta at 39 weeks of pregnancy), from the same patient, from an epigenomic point of view (by CpG islands methylation status). Human decidual natural killer cells (dNK) are a unique NK cell subset with a profound immunomodulatory potential [7]. dNK cells are rather different from other CD56 bright CD16 dim NK cells, and their specific function in uterine decidual stroma remains still unclear. NK cells are a rather heterogeneous group of innate lymphoid cells and have been traditionally related to anti tumor immune responses [8]. Most studies have referred to circulating peripheral blood NK cells (pNK cells) [9], which are quite different from tumor infiltrating NKcells (TINKs). In fact, there is increasing evidence that tumor microenvironment may induce a specific gene expression signature that renders TINK cells less tumoricidal and perhaps contribute to cancer progression [10, 11]. Uterine decidual NK cells (not the same as endometrial NK cells in non-pregnant uterus) are quite distinct from the rest [12]. Human CD56 bright CD16 dim NK cells accumulate in the maternal decidua during early pregnancy, making up 70% of tissue lymphocytes in first trimester, and then gradually decrease. They are found in direct contact with fetal trophoblasts. The specific functions of these decidual NK cells during early pregnancy are mostly unknown. Activated dNK cells can produce angiogenic factors (VEGF, ANG2) that promote spiral artery remodeling, secrete cytokines (GM-CSF, CSF-1, TNFα, INFγ, TGFβ, LIF, IL2, CXCL10, CXL12) that direct the migration and invasion of the trophoblast, and interact directly with trophoblast antigens by expressing surface receptors such as killer immunoglobulin-like receptor (KIR) and immunoglobulin-like transcript-2 (ILT2) [12].

Several mechanisms have been proposed to explain the inability of NK cells to kill the semi-allogeneic fetal cells. But perhaps their mission goes beyond, and there remains the intriguing possibility that at least some of these cells are paradoxically meant to feed and protect fetal cells from immune destruction. Hanna et al. [12] proposed, on the basis of genomic expression, that dNK cells but not peripheral blood-derived NK subsets, regulate trophoblast invasion both *in vitro* and *in vivo* by production of the interleukin-8 and interferon-inducible protein-10 chemokines. Furthermore, dNK cells are potent secretors of an array of angiogenic factors and induce vascular growth in the decidua. Notably, such functions are regulated by specific interactions between dNK-activating and dNK-inhibitory receptors and their ligands, uniquely expressed at the fetal-maternal interface. However, decidual NK cells do have potent intracellular cytotoxic machinery in some of their cytoplasmic granules, including powerful Granzyme/Perforins [12]. Contrary to standard beliefs, it remains possible but unproven that during embryo implantation and early placentation, their killer activity might not be directed against non-self trophoblast cells but against the mother´s own T-lymphocytes, which would specifically recognize them as non-self and eliminate the fetus. Thus, dNK cells might paradoxically protect trophoblast cells, while at the same time are capable of activating Tregs and inhibit Th-17 responses by releasing cytokines or direct cell contact [13, 14].

It could be expected that this very “special” decidual/placental microenvironment is most suitable for tempering antigenic challenges, and different cellular immune engagements are also bound to be “special”. All stromal cells (as in cancer) are expected to be potential “accomplices” of the physiological deceit or “betrayal” of the maternal immune system by the fetus, because the placenta was probably meant to protect the fetus rather than the mother.

How exactly do regulatory T cells work is still a matter of debate, not only in these contexts of feto-maternal tolerance and cancers, but also in many others [15]. Since 1988, when Göran Möller openly questioned the existence of “Suppressor T cells” [16] — without questioning the existence of suppressive phenomena or findings that T cells can mediate suppressive effects — considerable research efforts have added to the complexity of the subject. Unlike the little understood decidual or placental microenvironments, the best understood complex microenvironment where immune regulation and maturation occurs, is probably the thymus [17–22]. In the thymus, progenitor survival and lineage commitment require the TCR to interact with self-peptide MHC ligands on epithelial cells in the thymic cortex. These receptor-ligand interactions occur over a great range of affinities both because of the diversity of the TCR combining site amino acids, and because of the diversity of self-peptides displayed by each MHC allele. Quantifiable differences in TCR affinity for peptide-MHC (pMHC) complexes result in rather different selection outcomes, establishing the basis for positive and negative selection [17–22]. Weak TCR signals support positive selection, whereas strong agonist signals support the removal of a potentially self-reactive TCR through negative selection. Evolution also generated a system to allow thymocytes to ‘see’ a large array of self-peptides during development. This snapshot of ‘self’ happens in the medulla of the thymus via a specialized population of medullary thymic epithelial cells, mTECs [23]. These cells express a gene known as autoimmune regulator (AIRE) [21]. AIRE is a transcriptional regulator that permits expression of a diverse array of strictly tissue-restricted peripheral antigens within the thymus, to eliminate T cells with too strong affinity for any of these antigens [17–22]. Hence, thymocytes must express TCRs with very high sensitivity to peptide MHC (pMHC) signals received during maturation, to protect the host from allowing a self-reactive TCR to survive. Moreover, it is also true that thymocytes must express TCRs with the ability to transduce signals for very weak, low-affinity pMHC molecules to support positive selection. If they fail to recognize self pMHC and provide a TCR-specific survival signal, they undergo apoptosis via a process known as death by neglect. Therefore, a thymocyte’s fate is probably ultimately determined by its specificity and affinity for self pMHC. During carcinogenesis, according to the general immune vigilance concept, Tumor-associated antigens (TAAs), which could be upregulated self-antigens, altered-self antigens as a consequence of post-translational modifications or neoantigens generated by mutagenic events in tumor cells, may drive the early and rapid expansion of Treg cells [24]. Failure to kill tumor cells bearing such antigens presumably leads to escape from immune vigilance and to cancer progression. Several authors have published the detection in both human placenta and human cancers of specific gene products with immunoregulatory potential not usually expressed in normal somatic tissues [25–27]. Other differentially expressed genes identified by microarray gene profiling [28] have been associated with both preeclampsia and cancer growth, including several regulators of angiogenesis and immune responses. Finally, some recent work by Jasti et al. [29] provides evidence that immune response to a model shared placenta/tumor-associated antigen reduces cancer risk in parous mice. In this elegant pre-clinical model chicken ovalbumin (OVA) was used as a surrogate shared placenta/tumor antigen and OVA-bred mice had prolonged survival as compared to virgin mice: the progression of tumors was delayed in comparison to wild-type bred and virgin females. Paternally inherited OVA antigen elicited a CD8+ T cell response during pregnancy that was associated with delayed growth of OVA-expressing tumors following pregnancy. These results suggest a possible role of antigen-specific T cells in protecting parous females against tumors bearing shared placental/tumor antigens. Thus feto-maternal tolerance and cancer escape from immune vigilance are unlikely to be due to the action or activities of single isolated cell types or gene products, but of complex interactions in the tissue microenvironment of several cell types and biological regulatory peptides and molecules.

## RESULTS

**Figure 1:**
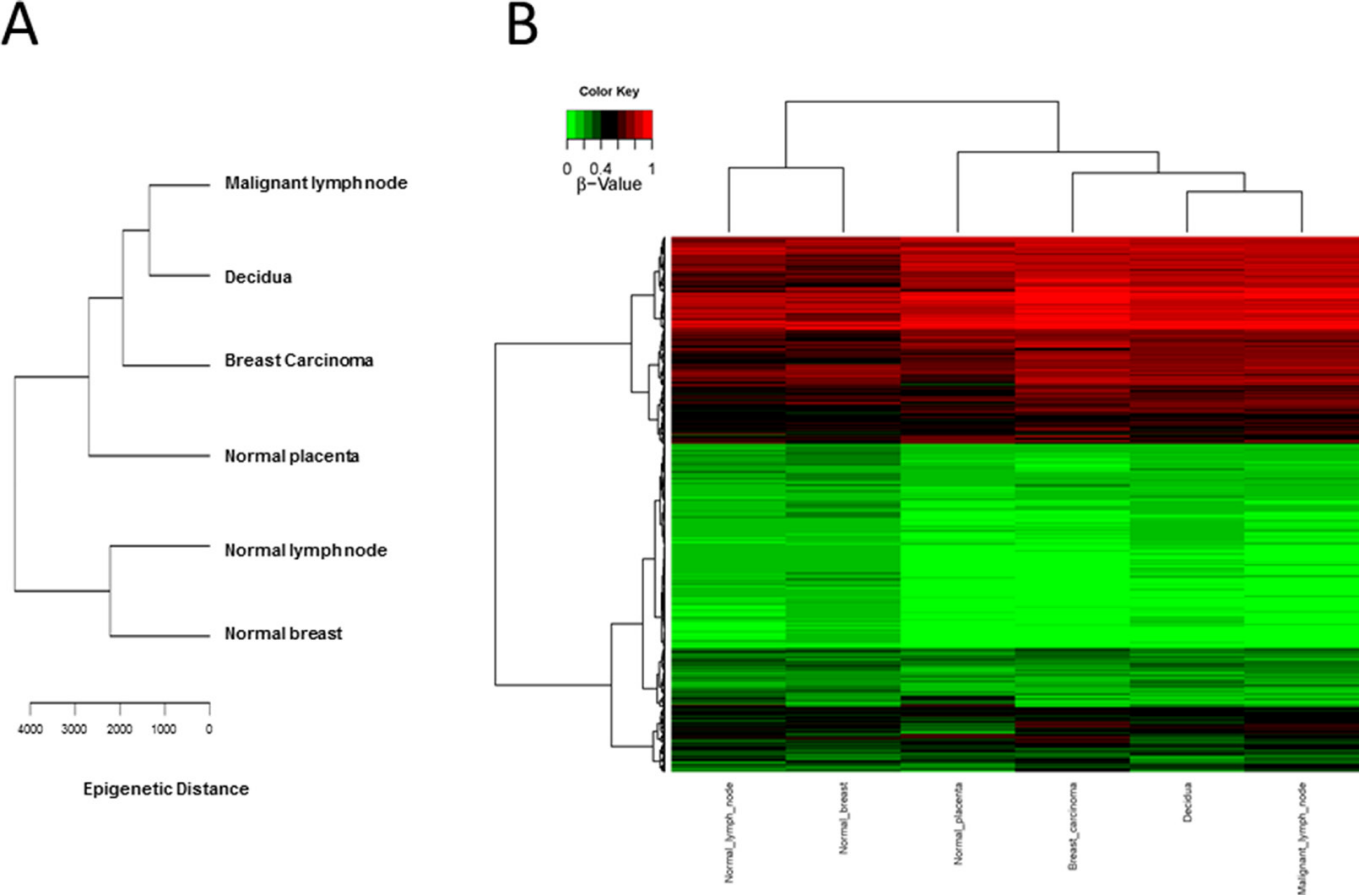
Unsupervised CpG island methylation (**A**) Schematic diagram of hierarchical clustering of the different tissues (malignant lymph node, decidua, breast carcinoma, normal placenta, normal lymph node and normal breast) taking all of the values of CpG islands from Supplemental Table 1 and calculating the “epigenetic distance” between the different tissues. Malignant tissues (breast cancer and metastatic lymph node) cluster more together with decidua/placenta tissue, as compared to normal lymph node and normal breast. (**B**) Hierarchical clustering HeatMap of the different tissues (malignant lymph node, decidua, breast carcinoma, normal placenta, normal lymph node and normal breast) colour coded (red: non-methylated; green: methylated).

**Figure 2:**
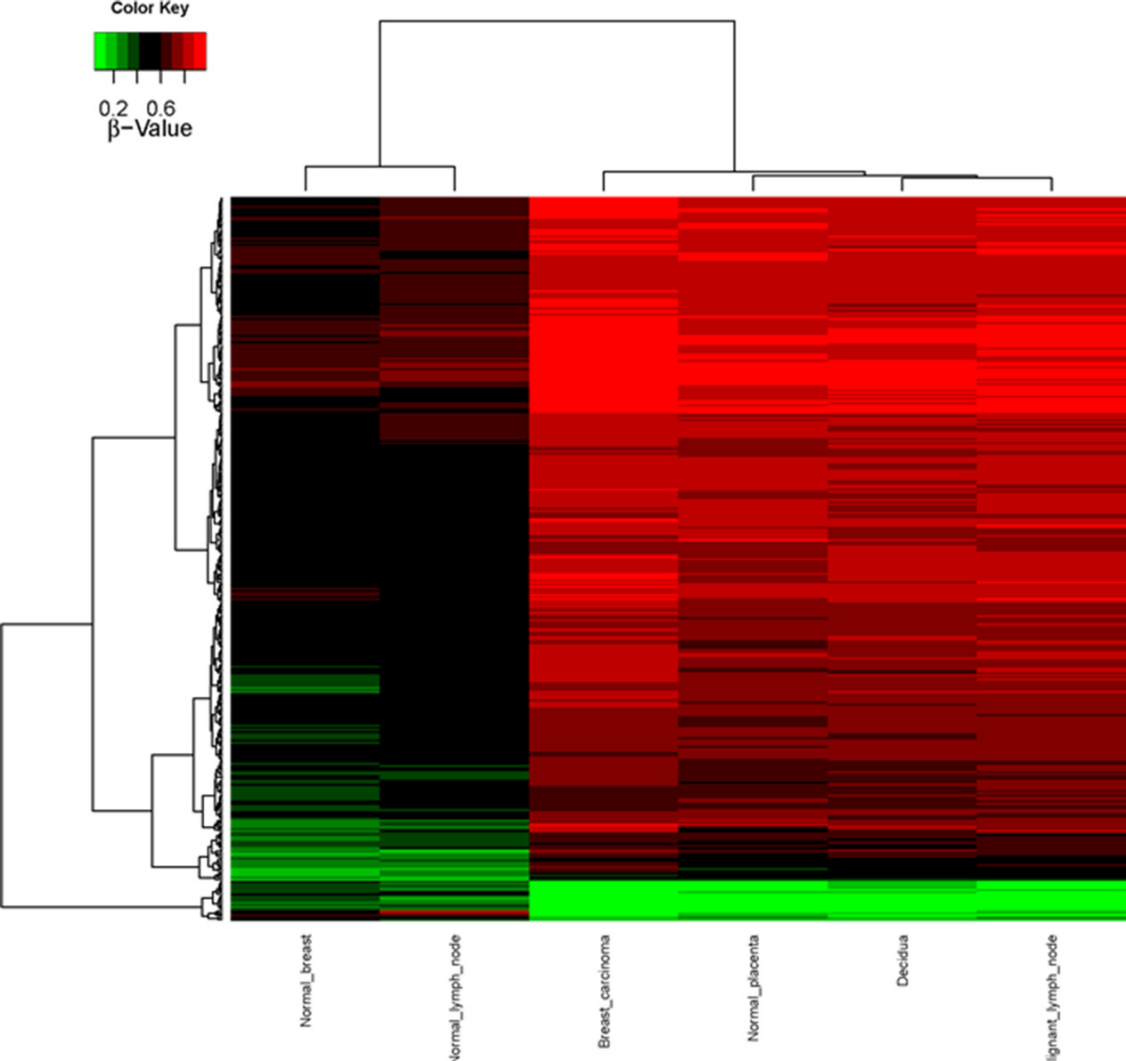
Supervised CpG island methylation hierarchical clustering heat-map Heatmap representation of the 343 selected features (rows) resulting from analysis of the different biopsies of the patient (columns), showing a differential methylation. Red denotes hypermethylation, green hypomethylation.

CpG methylation patterns were analyzed in all six samples. The methylation average for each CpG island was calculated for each of the tissue samples. Then, CpG islands with different methylation patterns between “self” (normal breast and normal lymph node) and “non-self” (breast cancer, metastatic lymph node as well as decidua and placenta) were searched for as described in Methods. Both supervised and unsupervised hierarchical clustering (Figures 1 and 2) confirmed a much closer relationship in CpG island methylation patterns between malignant (primary breast cancer and metastatic LN) and placental/decidual tissues, than between malignant tissues and their normal counterparts (normal breast and normal LN). Full methylation status in all genes analyzed are shown in Supplementary Table 1.

**Figure 3:**
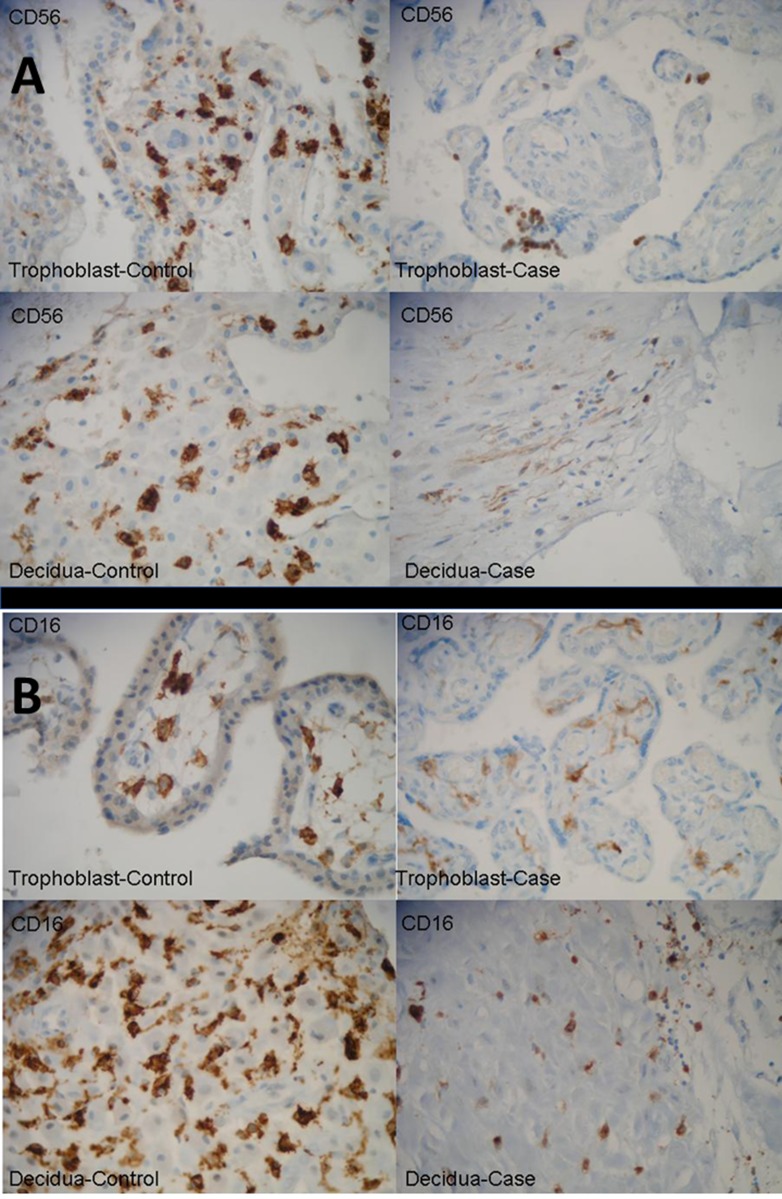
Representative tissues from decidua and placenta from our clinical case, at week 39 of pregnancy, and (named “controls” for illustrative purposes only) unrelated abortions secondary to embryonic natural death in first few weeks of pregnancy, immuno stained with CD56 and CD16 illustrating different tissue densities of dNK-cells Spontaneous abortions materials were used to identify NK cells next to trophoblasts but not for any specific comparison with our clinical and pathological case. (**A**) CD 56 immunostains strongly positive in abortion cases (“controls”) chorial villi (×400) and decidua (×400) (Top and Bottom Left), and weakly positive in case villi (×400) and case decidua (×400) (Top and Bottom Right). (**B**) CD 16 immunostains strongly positive in abortion cases (“controls”) chorial villi (×400) and decidua (×400) (Top and Bottom Left), and weakly positive in case villi (×400) and case decidua (×400) (Top and Bottom Right). No appreciable CD56 or CD16 staining was found in the primary breast cancer (and this is why these data are not shown).

With regards to histopathology and immunohistochemistry (IHC), (Figure 3) we did not find significant numbers of CD56+ or CD16+ NK subsets in our patient’s primary breast cancer (lobular breast carcinoma stage pT2 pN2a Mo, luminal-A, with low Ki67 positivity), but found very significant IHC staining of CD56++ and CD16++ cells in placenta and decidua tissue from unrelated abortions (not used as “controls”, but to look for NK cells in “early” placental tissues) at 6–10 weeks of gestation secondary to embryonic natural death. This confirms the abundance of dNK cells in early pregnancy microenvironments. Some positive but much weaker staining was also found in the decidua of the 39-weeks pregnant uterus of our clinical case, (Figure 3A and 3B). In our placental/decidual materials from spontaneous abortions in first trimester, trophoblast cells surrounded by lymphocytes with NK characteristics and CD56++ or CD16++ staining were abundant. But it is unclear if these cells were truly part of the physiological normal placental tissues in early pregnancy, or consequence of the pathological process which led to the death of the embryo and to abortion. We did not have liquid or fresh frozen samples from our clinical case (or controls), and we were unable to perform FACS analysis of “bright versus dim” NK cellular subsets on these samples.

**Figure 4:**
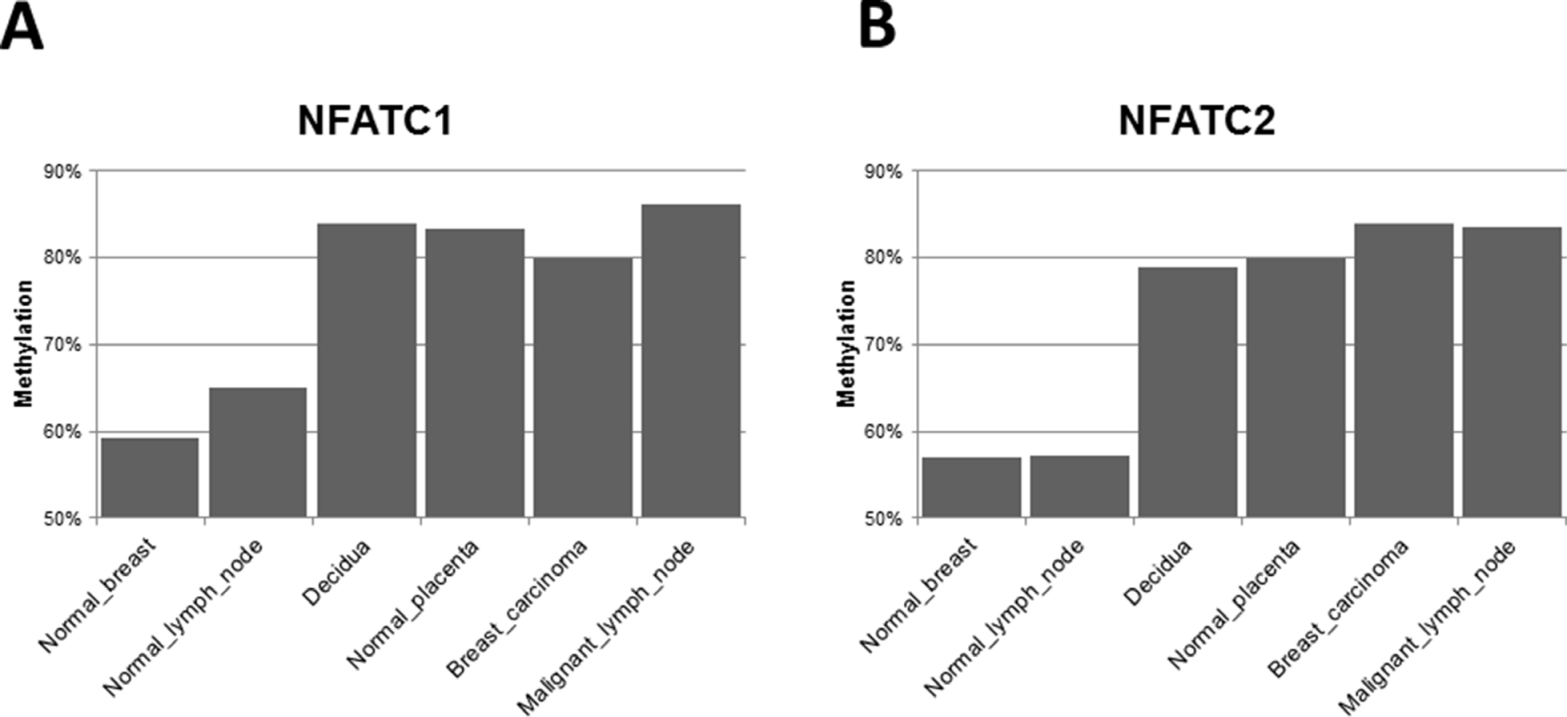
NFATc (nuclear factor of activated T cells) genes 1 and 2 CpG islands methylation patterns differentially expressed between “self” and “non-self” tissues. CpG island methylation level of the NFATC1 (**A**) and NFATC2 (**B**) genes, showing differential methylation between normal breast and lymph node biopsies in comparison with decidua, normal placenta, breast carcinoma and malignant lymph node.

Several important regulatory genes related to the immune response, angiogenesis and inflammation are differentially hypermethylated in their CpG regulatory DNA islands in both malignant (primary breast LBC and metastatic LN) and placental tissues (Supplementary Table 2), as compared to normal breast and LN. Of these, “nuclear factors of activated T cells” (NFAT) genes are some of the most interesting (Figure 4), with some of the clearest variations in expression between normal breast and LN tissues (with lower CpG island methylation) and placental/malignant tissues (with considerably higher CpG methylation). NFATp (also named NFATc2) and NFATc1 are two genes whose CpG island methylation patterns are more clearly differentially methylated in the “non-self” tissues of our clinical case (decidual/placenta and malignant breast and metastatic LN), as opposed to the “self tissues” (normal breast and normal LN) (Figure 4). The nuclear translocation of NFATc2 (also known as NFATp or pre-existing) is regulated by calcium and calcineurin, and inhibited by potent immunosuppressive drugs like cyclosporin A and tacrolimus. Methylation of CpG islands in DNA regulatory regions usually leads to under expression of the corresponding genes. But, in spite of similar levels of CpG island methylation in the “non-self” tissues (Figure 4), these transcription factor genes are expressed rather differently. For example, NFATc 1 and 2 gene expression (as detected by Nanostring genomics, see Supplementary Table 2 in ref. [6]) is decreased by approximately 50% in the primary breast cancer as compared to normal breast, in accordance with much higher CpG island methylation (Figure 4). But, surprisingly, these genes are overexpressed (more than double) in the metastatic LN than in the normal LN, even if their CpG islands methylation patterns are similar to the primary breast cancer tissue. These discrepancies suggest that the two microenvironments (primary breast cancer and metastatic LN) differ, and probably diverse regulatory gene expression mechanisms, other than simply CpG island methylation, are at work in the metastatic lymph nodes.

## DISCUSSION

Our analysis of CpG island methylation patterns and differential profiles in “self” (normal breast and normal LN) versus “non-self” (placenta/decidua and malignant breast or LN) confirm our genomics findings [6] of rather similar patterns of gene expression between uterine microenvironment in pregnancy and cancer. In fact, both unsupervised and supervised hierarchical clustering (genomic and epigenomic) confirm closer clustering patterns between decidua/placenta and malignant tissues, than between normal breast and malignant breast, or normal LN and malignant LN. Supplementary Tables 1 and 2 provide detailed information on specific genes and CpG islands methylation patterns.

Our genomic analysis (using Nanostring PanCancer Immune Kit) showed that 258 immune related genes were upregulated and 44 genes downregulated in breast tumor versus normal breast tissue. And 178 genes were upregulated while 146 genes downregulated in tumor bearing lymph node versus non-involved lymph node [6]. Many of the genes and promoter regions are similarly differentially methylated at CpG islands in these same tissue samples, and overexpressed or underexpressed in the same fashion. This is true also for immune-related genes and pro-tolerogenic and angiogenic proteins. These complex results in one single clinical case need confirmation in further studies (both clinical and pre-clinical), but strongly suggest that several cell types in the microenvironment are involved in feto-maternal tolerance and possibly also in cancer immune escape. Among the main cell types in the stroma, six of them are associated with many of the gene products over- or underexpressed or similarly methylated at CpG islands (see genomic Supplementary Tables 1 and 2 in ref. [6], and epigenomic Supplementary Tables 1 and 2 in this paper): macrophages, dendritic cells, myeloid-derived suppressor cells, B-lymphocytes, T-effector cells, and NK cells. Although we found no evidence of significant dNK cells in the primary breast cancer of our patient, and only limited presence in decidua/placenta at 39-weeks of gestation, there are a number of interesting publications that suggest a very special role for these NK subtypes, both in pregnant uterine and malignant microenvironment. If dNK cells do play a role in protecting the fetus from maternal immune attack, they should do so very early on in pregnancy (when their cellular NK density is at its peak in decidua/placental tissue, and clearly before immunological rejection of the fetus). Similarly, during carcinogenesis, if similar NK cells play a role in eliminating anti-tumor endogenous T cells, this must happen early on in cancer development, before the time of malignant cell escape from immune vigilance. Although the presence of Tumor-Infiltrating Natural Killer (TINK) within a tumor bed may be indicative of an ongoing immune response toward the tumor, and often carries a better prognosis, many studies have paradoxically shown that an intense NK infiltration can be associated with advanced disease, and may even facilitate cancer development [30, 31]. The exact role of the tumor infiltrating NK cells and the correlation between their presence and poor prognosis remains a matter of controversial debate. Levi et al. [32] have elegantly demonstrated by IHC and FACS (fluorescent activated cell sorting) a significant enrichment of a CD56 brigh CD16 dim NK cells within several tumors. These NK cells express several dNK-like markers, including VEGF among others. Curiel et al. [31] have previously demonstrated a specific recruitment of regulatory T cells (Treg) into ovarian cancer tissue. These cells have inhibitory activities toward CD4 helper and CD8 cytotoxic T cells. These authors further established a correlation between the number of tumor-derived Treg cells and patient’s survival. Hence, attracting these regulatory cells to tumor beds may represent a mechanism by which tumors may evade the host immune response. Levi et al. [32] characterized TINK cell subsets and demonstrated that similarly to early decidua, there is a significant enrichment of the CD56 brigh CD16 dim NK subset, and that these cells express the pro-angiogenic factor VEGF, making cancer microenvironments somewhat similar to decidual microenvironment [32].

We did not find appreciable numbers of CD56+ or CD16+ NK subsets in our patient’s primary breast cancer (LBC, pT2 pN2a Mo), with immunohistochemistry (IHC) compatible with “Luminal A subtype” (data not shown because IHC for CD56 and CD16 was essentially negative in the primary tumor). We found very significant IHC staining of CD56++ and CD16++ cells in placenta and decidua tissue from unrelated abortions at 6–10 weeks of gestation, secondary to natural embryonic death. These results confirm the abundance of dNK cells in early pregnancy microenvironments, although in these cases the presence of these NK cells could also be part of the pathological process that led to the natural death of the embryo. There were also frequent trophoblasts surrounded by lymphocytes with NK characteristics and CD56++ staining. The decidua of our 39-weeks pregnant uterus showed a positive but relatively weak staining with CD56 and CD16 IHC (Figure 3A and 3B). CD56 (also known as Neural cell adhesion molecules, NCAM, or Leu19) is a membrane glycoprotein homophilic cell adhesion molecule, that was originally isolated in relationship with its role in neural cell adhesion. But it is also a good marker of human NK cells, as during hematopoietic differentiation CD56 is the prototypic marker of NK cells, though also present on a subset of CD4+ T cells and some CD8+ T cells. CD16 has been identified as Fc receptors FcγRIIIa (CD16a) and FcγRIIIb (CD16b). These receptors bind to the Fc portion of IgG antibodies, which then activate the NK cell for antibody-dependent cell-mediated cytotoxicity [33].

Unlike peripheral blood pNK cells, dNK cells are only weakly cytotoxic against standard cancer cell lines (e.g., K562), and do not normally kill trophoblast cells [34–36]. This is intriguing, as dNK cells are large lymphocytes with prominent cytoplasmic granules containing perforin, granzymes, and granulysin, which suggests that other, noncytolytic functions of these proteins are important [37–39]. Although dNK cells do not normally kill, they might become cytolytic in certain situations, such as CMV infection, in which cellular stress is detected by the NKG2D receptor [40]. Human dNK cells are confined to the mucosa, endometrium and decidua, and they can probably derive from several pools [41, 42], like pre-existing resident endometrial NK cells (eNK), peripheral blood NK precursors or uterine CD34+ cells in the human endometrial mucosa. dNK cells function has been proposed to be to cooperate with trophoblast cells to guarantee correct arterial remodeling, ensuring the supply line to the growing fetus. But it is also possible (yet speculative) that relevant dNK cells in the decidual microenvironment might also attract and selectively destroy some immune competent T-effector cells, from the idiotype/anti-idiotype maternal pool, with affinity TCR for at least some but not all paternal antigens. Perhaps similarly, tumoral dNK-like cells present at the most vulnerable time of carcinogenesis (when immune escape occurs) could destroy “*in situ*”, before they expand in numbers, the relevant immune competent clones of some host T-effector cells potentially capable of tumor antigen recognition. As a result, the immune system of the host is unable to suppress malignant growth, and local tissue invasion or metastasis occur. Following this critical phenomenon, residual TINKs may persist in variable numbers in tumor microenvironments [32], or perhaps disappear altogether, as in our clinical case and other cancers. We are aware that we do not provide direct evidence for this particular hypothesis, may be because our tissue samples could have “well past” that presumably “critical window of time” for both these events, namely early trophoblast implantation or early carcinogenesis and immune vigilance escape. Besides the already mentioned fact that T cells, antigen-presenting cells and possibly B cells are also likely to play key roles in both feto-maternal tolerance and cancer escape from immune vigilance. However, dNK cells and TINKs are usually classified as part of the innate immune system, an evolutionary older defense strategy than the adaptive immune system, and this concept can also have important evolutionary implications. For example, it remains unknown why most epithelial cancers are more aggressive in their malignant properties (local tissue invasion and metastasis) in mammals than in invertebrates, or indeed in other vertebrate animals like fish, reptiles or even the most recently evolved birds [43]. This more aggressive and potentially lethal cancer behavior is perhaps surprising in organisms such as mammals, with a far more sophisticated and powerful immunological network than non-mammals. Unless they can ectopically and inappropiately express key immune vigilance escape gene programs, evolved precisely from mammals, to allow for feto-maternal tolerance. Males do obviously not have uterine decidual tissues, but perhaps at least some of their regulatory immune cells (NK cells or T cells or others) can be induced (at some specific time points during carcinogenesis) to ectopically express those “placental gene programs” responsible for our proposed “allo-selective T-effector cell neutralization”, abrogating any remaining immune vigilance role. This does not rule out other T cell regulatory mechanisms as recently shown by Jasti el al. [29] who, in an elegant mouse model, have shown that paternally inherited OVA antigen elicited a CD8+ T cell response during pregnancy that was associated with delayed growth of OVA-expressing tumors following pregnancy. These results suggest a possible role of antigen-specific T cells in protecting parous females against tumors bearing shared placental/tumor antigens.

Co-evolution of Human Leukocyte Antigen (HLA) Class I Ligands with Killer-Cell Immunoglobulin-like Receptors (KIRs, like those present in dNK cells), during the emergence of founding populations of modern humans (Homo Sapiens Sapiens) in Subsaharan Africa, might have driven specific genomic placental feto-maternal tolerance immune mechanisms while protecting the fetus from important endemic parasytes, like pathogenic malarial *Plasmodium falciparum* [44]. Some of the potential peptides or proteoglycans involved might perhaps be also related to recently described chondroitin sulphate CS-oncofetal antigens, which have been identified in both cancer stromal microenvironments and on the membrane surface of some trophoblast cells [45]. This CS-antigen expression in the extra cellular matrix (ECM) is of particular interest with regard to immune surveillance, because ECM-associated glycosaminoglycans (GAGs) have been affiliated with the immune process for decades [46]. Using a specific placental glycan-binding malaria protein, Salanti et al. [45]. have demonstrated that placental-like glycans are widely expressed in human tumors, with absent-to-low expression in normal tissues other than placenta. By conjugation of cytotoxic compounds to this protein, they recently demonstrated its capacity to specifically target cancer cells and block tumor growth “*in vivo*”. The “immunological synapse” in the decidual microenvironment between dNK cells and trophoblast cells — or the one that might occur at some early point during carcinogenesis in tumor microenvironment between TINK-like cells and tumor cells — might have evolutionary conserved molecular mechanisms, implicating complex and specific recognition patterns at the “synapse” between glycoproteins and specific immune peptides or proteins [47–52].

On balance, we provide circumstantial genomic and epigenomic evidence for a previously unreported biological phenomenon: cancers may somehow “hijack” gene programs evolved over millions of years to allow for feto-maternal tolerance (in placental mammals), in order to escape from immune vigilance and spread as malignant local invasive cancer or metastasis. Exactly how they do it, or what are the precise molecular and cellular mechanisms by which these ectopically expressed genes facilitate escape from immune vigilance, is not known, and we cannot prove or disprove them. Other alternative or complementary mechanisms to the main one proposed here are, of course, also possible. Human cancers are genetically and epigenetically heterogeneous from the moment they are clinically diagnosed, so that even effective chemotherapies often merely select preexistent chemo-resistant cellular clones. But it is well known that most cancers (and their metastasis) “evolve” and may accumulate “new mutations” and “new antigenic epitopes” as they progress, so that their local microenvironment should maintain a pro-tolerogenic milieu (locally and perhaps systemically) until the cancer is cured or the patient dies. Once cancer microenvironments have adopted the placental pro-tolerogenic gene programs, they must be capable of using them to escape from immune control (whatever the precise mechanisms), at each successive round of new tumor antigen expression by cancer cells or evolution of neoantigen landscapes.

Nielsen et al. [53] found that both subsets of human NK cells, CD56dim and CD56bright, kill activated CD4+T cells. Although CD56dim NK cells are typically cited as being the more cytotoxic subset, due to higher levels of perforin, granzymes and cytolytic granules, other studies have shown that following activation, CD56 bright NK cells are equally, if not more, cytotoxic and can use other powerful cell killing pathways like, for example, TRAIL [54–57]. Nielsen’s study confirms that both CD56 NK subsets are equally cytotoxic following IL-2 activation, and are equally efficient at T cell killing if these T cells are already activated and not resting [53]. Degranulation by NK cells towards activated CD4+ T cells was enhanced by some cytokines or chemokines and not by others, depending on specific repertoires of activating (NKG2D and others) or inhibitory receptors (like inhibitory killer cell immunoglobulin-like receptors iKIR) and specific ligands. It also depends on the fine tuning of “Dim” or ”Bright” phenotypes for both CD56 and CD16 membrane antigens. In the case of feto-maternal antigens on trophoblasts at the placenta/decidual level, and to our knowledge, no one has yet shown selective NK killing of those specific maternal CD4+ T cells activated by paternal allo-antigens. Regulation of immune response in NK cells has been shown [58]. upon CD16 ligand binding, and it is also induced by the activation and expression of the so-called “nuclear factors of activated T cells” (NFATc1 and NFATc2), which are two genes whose CpG island methylation patterns are more clearly differentially activated in the “non-self” tissues of our patient (decidual/placenta and malignant breast and metastatic LN), as opposed to the “self tissues” (normal breast and normal LN) (Figure 4). Several important regulatory genes of the immune response, angiogenesis and inflammation are differentially hypermethylated in their CpG regulatory DNA islands, in both malignant and placental tissues (Supplementary Table 2). Of these, NFAT genes are some of the most interesting and with the clearest variation in CpG islands methylation between normal breast and LN tissues (with low CpG island methylation) and placental/malignant tissues (considerably higher CpG methylation). NFAT proteins were first discovered in T cells in the 1980s, as transcriptional activators of interleukin-2 (IL2), a key regulator of T cell immune response, that at the time was also successfully introduced into practice as immune therapy for some renal cancers and malignant melanoma [59, 60]. Treatments with Rhu-IL2 were reasonably active in some malignancies but toxic, often requiring admission to intensive care unit (ICU) for cytokine-cascade related shock. The potent immunosuppressants cyclosporine A and tacrolimus inhibit the NFAT pathway to reduce rejection in patients receiving organ transplantation [59, 60]. Thus, the markedly increased CpG island methylation (and decreased gene expression) of NFATc genes 1 and 2 (Figure 4 and Supplementary Table 2 in ref. [6]), that we have found in the primary breast cancer of our patient (but not in the metastatic LN), could result in a potent localized “cyclosporine-A like immunosuppressive effect” to the benefit of cancer cells in the primary cancer stroma but not in the metastatic LN.

With regards to oncogenes and tumor suppressor genes, NFATc1 signaling has been associated with changes in the malignant properties of transformed cells by different mechanisms, at times contradictory [61, 75]. In general, the overexpression of these genes has been reported as pro-oncogenic, though in some experimental models they have paradoxically tumor suppressor effects [62]. These apparently contradictory effects are probably due to the weak DNA binding property of NFAT, which requires that NFAT partners or couples molecularly with other factors to execute transcription regulation, like GATA to control heart development, FOXP3 to regulate immune tolerance, or AP-1 to trigger T cell response. Finally, NAFTc genes 1 and 2 have been known to be key factors as “positive regulators” to both vertebrates evolution and T and B cell activation and differentiation [63, 64], while their effect in NK cell antitumor reactivity has been recently suggested to be “negative” [65].

It is now widely accepted that NK cells can shape the adaptive immune system by influencing T cells in different stages of their lifespan. During T cell priming, NK cells indirectly alter T cell responses by affecting dendritic cells (DCs), another major potential cellular player in this context. Reciprocally, DCs are also able to modulate NK cells and this bidirectional interaction affects the emerging T cell response [66]. Human CD56 bright NK cell subset is most prominent in peripheral lymphoid òrgans, compared with the CD56 dim subset which mostly circulates. CD56 bright cells derived from healthy individuals can suppress autologous CD4 T cell proliferation under inflammatory conditions [67, 68]. These fundamental and evolutionary ancient processes are probably relevant to our hypothesis on placental and malignant regulatory microenvironments, and deserve further scrutiny in the context of both feto-maternal tolerance and immune escape during carcinogenesis. It seems plausible to speculate that during the evolution of placenta (presumably from non placental mammals over 100 million years ago) the uterine microenvironment evolved mechanisms of feto-maternal tolerance by developing different ways to limit autoimmunity as, for example, in chronic viral infections. Mechanisms like NK cells control of CD4+ T cell responses, or ways of modulating CD8+ T cell responses and possibly also B cell responses.

Our two studies (genomic and epigenomic) on a single pregnant breast cancer patient, provide us with a first complex and data-dense glimpse on the molecular parallelism between placental and cancer microenvironments. But they are only a «photo» at one particular time and of one particular case. Only when we will have the full “movie-picture”, in different cases and times, from different experimental and clinical models, we should be able to fully comprehend the nature of the incredible evolutionary jump that gave birth to placental mammals over 100 million years ago [69, 70], and perhaps also to the higher risk of malignant epithelial cancers. In this respect, it is intriguing that the two surviving species of non placental mammals (Australian Echidna and Platypus Ornithorhyncus) have breast tissue (ducts and lobules) but seldom (if ever) are report to die naturally from breast cancer, and although they can suffer from neoplasia, aggressive metastatic epithelial carcinomas are uncommon as a cause of death, even if their life span can be considerable (up to 45 years in short beaked echidnas) [71, 72].

## MATERIALS AND METHODS

### Clinical case

In 2000, a 32 years-old pregnant woman with a palpable breast lump underwent delivery of non-identical twins by C-section at week 39 of a normally developing pregnancy. A total hysterectomy was performed during C-section to control for profuse bleeding, caused by attempting manual separation of the complex membranous placenta with velamentous umbilical cord insertion. Following histological confirmation of the malignant nature of the breast nodule, the patient underwent right mastectomy and axillary lymphadenectomy (with removal of 15 lymph nodes). The tumor was reported as invasive lobular breast carcinoma (LBC) stage pT2 pN2a Mo (5/15 axillary lymph nodes with metastatic disease), with immunohistochemistry (IHC) compatible with “Luminal A subtype” (ER+, PR+, HER-2 negative and Ki67 5% positivity) and histological evidence of vascular and lymphatic invasion by cancer cells, and some *in situ* lobular carcinoma as background component. The patient received standard of care adjuvant chemotherapy, loco regional radiotherapy and adjuvant hormone therapy (tamoxifen for five years) and was followed for over ten years without evidence of loco-regional or distant recurrence.

### Histopathology

Representative tissues from decidua and placenta from this clinical case, and unrelated first trimester abortions, secondary to embryonic natural death, were fixed in 10% formalin buffered at room temperature for 24 hours. Samples were paraffin embedded, cut into 5 µm-thick sections and stained according to hematoxilin-eosin standard techniques. Additional sections were stained with immunohistochemical procedures for detection of CD56 (Cell Marque clone MRQ-42), and CD16 (Cell Marque clone SP175).

### Methylation profiles

Per sample, one section of 10 μm from the FFPE biopsy blocks were hematoxylin-eosin stained and reviewed by a pathologist to mark tumoral tissue areas. Between 6 and 8 sections of 10 μm were used to isolate genomic DNA, and a macroscopic dissection was performed on those previously annotated regions. DNA extraction was done following standard indications of manufacturer’s DNA isolation kit (E.Z.N.A. FFPE DNA isolation kit, Omega Bio-tek, Norcross, GA, USA). Obtained gDNAs were quantified using a fluorimetric method (Qubit, ThermoFisher Scientific, Waltham, MA, USA). Bisulfite conversion of 250 ng of genomic DNA was performed using EZ DNA methylation kit (Zymo Research. Irvine), following manufacturer’s instructions. Two hundred ng of bisulfite converted DNA were used for hybridization on the Illumina Infinium MethylationEPIC BeadChip (Illumina, Inc. San Diego), following previously published description [73, 74]. Fluorescent signals from the microarray were measured with a HiScan scanner (Illumina, Inc. San Diego) using iScan Control Software (V 3.3.29).

### Methylation analysis

Microarray intensities were normalized against control probes of the microarray, using the mini package (preprocess Illumina), and background level adjusted. All beta values with an associated *p*-value greater or equal to 0.01 were removed from the analysis. Average methylation was computed per CpG island using the manufacturer’s annotation file for the MethylationEPIC BeadChip. For genes without an annotated CpG Island, the average methylation level of the promoter (CpGs annotated to be located on TSS200, TSS1500, 5’UTR or 1stExon) was computed instead. Analysis of the average methylation variance for each CpG island / Promoter was done differentiating among the 3 samples groups (1: Normal breast and lymph node; 2: Decidua and normal placenta; 3: Breast carcinoma and malignant lymph node). The Tukey’s ‘Honest Significant Difference’ method was applied to calculate adjusted *p*-values. The following criteria were used to select differentially methylated CpG islands: CpGs islands / Promoters whose absolute methylation difference was greater or equal to 0.2 between groups 1–2 and 1–3 and whose associated FDR *p*-value were lower or equal to 0.05. All hierarchical clustering were performed over the Manhattan distances aggregated by ward’s linkage. Assessment of uncertainty of the hierarchical clustering was performed using Pvclust package (nboot = 1000) under R statistical environment.

## SUPPLEMENTARY MATERIALS TABLES







## Abbreviations

LN: lymph node
T-reg: regulatory T cell
EVT: extravillous trophoblast
NK: natural killer
TINK: tumor infiltrating NK cells
VEGF: vascular endothelial growth factor
TCR: T cell receptor
MHC: major histocompatibility complex
IHC: immunohistochemistry
LBC: lobular breast carcinoma

## References

[1] Clarke CA (1968). Immunology of pregnancy: significance of blood group incompatibility between mother and foetus. Proc R Soc Med.

[2] Staun-Ram E, Shalev E (2005). Human trophoblast function during the implantation process. Reprod Biol Endocrinol RBE.

[3] Lunghi L, Ferretti ME, Medici S, Biondi C, Vesce F (2007). Control of human trophoblast function. Reprod Biol Endocrinol RBE.

[4] Holtan SG, Creedon DJ, Haluska P, Markovic SN (2009). Cancer and pregnancy: parallels in growth, invasion, and immune modulation and implications for cancer therapeutic agents. Mayo Clin Proc.

[5] Svensson-Arvelund J, Mehta RB, Lindau R, Mirrasekhian E, Rodriguez-Martinez H, Berg G, Lash GE, Jenmalm MC, Ernerudh J (2015). The human fetal placenta promotes tolerance against the semiallogeneic fetus by inducing regulatory T cells and homeostatic M2 macrophages. J Immunol.

[6] Bronchud MH, Tresserra F, Xu W, Warren S, Cusido M, Zantop B, Zenclussen AC, Cesano A (2016). Placental immune editing switch (PIES): learning about immunomodulatory pathways from a unique case report. Oncotarget.

[7] Koopman LA, Kopcow HD, Rybalov B, Boyson JE, Orange JS, Schatz F, Masch R, Lockwood CJ, Schachter AD, Park PJ, Strominger JL (2003). Human decidual natural killer cells are a unique NK cell subset with immunomodulatory potential. J Exp Med.

[8] Cheng M, Chen Y, Xiao W, Sun R, Tian Z (2013). NK cell-based immunotherapy for malignant diseases. Cell Mol Immunol.

[9] Imai K, Matsuyama S, Miyake S, Suga K, Nakachi K (2000). Natural cytotoxic activity of peripheral-blood lymphocytes and cancer incidence: an 11-year follow-up study of a general population. Lancet.

[10] Gillard-Bocquet M, Caer C, Cagnard N, Crozet L, Perez M, Fridman WH, Sautès-Fridman C, Cremer I (2013). Lung tumor microenvironment induces specific gene expression signature in intratumoral NK cells. Front Immunol.

[11] Vitale M, Cantoni C, Pietra G, Mingari MC, Moretta L (2014). Effect of tumor cells and tumor microenvironment on NK-cell function. Eur J Immunol.

[12] Hanna J, Goldman-Wohl D, Hamani Y, Avraham I, Greenfield C, Natanson-Yaron S, Prus D, Cohen-Daniel L, Arnon TI, Manaster I, Gazit R, Yutkin V, Benharroch D (2006). Decidual NK cells regulate key developmental processes at the human fetal-maternal interface. Nat Med.

[13] Fu B, Li X, Sun R, Tong X, Ling B, Tian Z, Wei H (2013). Natural killer cells promote immune tolerance by regulating inflammatory TH17 cells at the human maternal-fetal interface. Proc Natl Acad Sci U S A.

[14] Kopcow HD, Allan DSJ, Chen X, Rybalov B, Andzelm MM, Ge B, Strominger JL (2005). Human decidual NK cells form immature activating synapses and are not cytotoxic. Proc Natl Acad Sci USA.

[15] Corthay A (2009). How do regulatory T cells work?. Scand J Immunol.

[16] Möller G (1988). Do suppressor T cells exist?. Scand J Immunol.

[17] Starr TK, Jameson SC, Hogquist KA (2003). Positive and negative selection of T cells. Annu Rev Immunol.

[18] Klein L, Hinterberger M, Wirnsberger G, Kyewski B (2009). Antigen presentation in the thymus for positive selection and central tolerance induction. Nat Rev Immunol.

[19] Murata S, Sasaki K, Kishimoto T, Niwa SI, Hayashi H, Takahama Y, Tanaka K (2007). Regulation of CD8+ T cell development by thymus-specific proteasomes. Science.

[20] Gommeaux J, Grégoire C, Nguessan P, Richelme M, Malissen M, Guerder S, Malissen B, Carrier A (2009). Thymus-specific serine protease regulates positive selection of a subset of CD4+ thymocytes. Eur J Immunol.

[21] Anderson MS, Venanzi ES, Klein L, Chen Z, Berzins SP, Turley SJ, von Boehmer H, Bronson R, Dierich A, Benoist C, Mathis D (2002). Projection of an immunological self shadow within the thymus by the aire protein. Science.

[22] Davey GM, Schober SL, Endrizzi BT, Dutcher AK, Jameson SC, Hogquist KA (1998). Preselection thymocytes are more sensitive to T cell receptor stimulation than mature T cells. J Exp Med.

[23] Moran AE, Hogquist KA (2012). T cell receptor affinity in thymic development. Immunology.

[24] Liu C, Workman CJ, Vignali DAA (2016). Targeting regulatory T cells in tumors. FEBS J.

[25] Kojima K, Kanzaki H, Iwai M, Hatayama H, Fujimoto M, Narukawa S, Higuchi T, Kaneko Y, Mori T, Fujita J (1995). Expression of leukaemia inhibitory factor (LIF) receptor in human placenta: a possible role for LIF in the growth and differentiation of trophoblasts. Hum Reprod.

[26] Yuan H, Glazer R, Wang X, Jin L, Li J, Vijayendra N, Doodala V, Weiss S (2015). Placental protein-1 (plac1) modulates immune tolerance in mammary tumor cells. J Immunother Cancer.

[27] Halpern M, Zahalka MA, Traub L, Moroz C (2007). Antibodies to placental immunoregulatory ferritin with transfer of polyclonal lymphocytes arrest MCF-7 human breast cancer growth in a nude mouse model. Neoplasia N Y N.

[28] Louwen F, Muschol-Steinmetz C, Reinhard J, Reitter A, Yuan J (2012). A lesson for cancer research: placental microarray gene analysis in preeclampsia. Oncotarget.

[29] Jasti S, Farahbakhsh M, Nguyen S, Petroff BK, Petroff MG (2017). Immune response to a model shared placenta/tumor-associated antigen reduces cancer risk in parous mice. Biol Reprod.

[30] Georgiannos SN, Renaut A, Goode AW, Sheaff M (2003). The immunophenotype and activation status of the lymphocytic infiltrate in human breast cancers, the role of the major histocompatibility complex in cell-mediated immune mechanisms, and their association with prognostic indicators. Surgery.

[31] Curiel TJ, Coukos G, Zou L, Alvarez X, Cheng P, Mottram P, Evdemon-Hogan M, Conejo-Garcia JR, Zhang L, Burow M, Zhu Y, Wei S, Kryczek I (2004). Specific recruitment of regulatory T cells in ovarian carcinoma fosters immune privilege and predicts reduced survival. Nat Med.

[32] Levi I, Amsalem H, Nissan A, Darash-Yahana M, Peretz T, Mandelboim O, Rachmilewitz J (2015). Characterization of tumor infiltrating natural killer cell subset. Oncotarget.

[33] Janeway C

[34] Ferry BL, Starkey PM, Sargent IL, Watt GM, Jackson M, Redman CW (1990). Cell populations in the human early pregnancy decidua: natural killer activity and response to interleukin-2 of CD56-positive large granular lymphocytes. Immunology.

[35] King A, Birkby C, Loke YW (1989). Early human decidual cells exhibit NK activity against the K562 cell line but not against first trimester trophoblast. Cell Immunol.

[36] Ritson A, Bulmer JN (1989). Isolation and functional studies of granulated lymphocytes in first trimester human decidua. Clin Exp Immunol.

[37] King A, Wooding P, Gardner L, Loke YW (1993). Expression of perforin, granzyme A and TIA-1 by human uterine CD56+ NK cells implies they are activated and capable of effector functions. Hum Reprod.

[38] Chen Z, Zhang J, Hatta K, Lima PDA, Yadi H, Colucci F, Yamada AT, Croy BA (2012). DBA-lectin reactivity defines mouse uterine natural killer cell subsets with biased gene expression. Biol Reprod.

[39] Vujaklija DV, Gulic T, Sucic S, Nagata K, Ogawa K, Laskarin G, Saito S, Haller H, Rukavina D (2011). First trimester pregnancy decidual natural killer cells contain and spontaneously release high quantities of granulysin. Am J Reprod Immunol.

[40] Siewiera J, El Costa H, Tabiasco J, Berrebi A, Cartron G, Le Bouteiller P, Bouteiller P, Jabrane-Ferrat N (2013). Human cytomegalovirus infection elicits new decidual natural killer cell effector functions. PLoS Pathog.

[41] Vacca P, Vitale C, Montaldo E, Conte R, Cantoni C, Fulcheri E, Darretta V, Moretta L, Mingari MC (2011). CD34+ hematopoietic precursors are present in human decidua and differentiate into natural killer cells upon interaction with stromal cells. Proc Natl Acad Sci U S A.

[42] Carlino C, Stabile H, Morrone S, Bulla R, Soriani A, Agostinis C, Bossi F, Mocci C, Sarazani F, Tedesco F, Santoni A, Gismondi A (2008). Recruitment of circulating NK cells through decidual tissues: a possible mechanism controlling NK cell accumulation in the uterus during early pregnancy. Blood.

[43] Kaiser HE (1989). Comparative aspects of tumor development. In: Cancer Growth and Progression.

[44] Norman PJ, Hollenbach JA, Nemat-Gorgani N, Guethlein LA, Hilton HG, Pando MJ, Koram KA, Riley EM, Abi-Rached L, Parham P (2013). Co-evolution of human leukocyte antigen (HLA) class I ligands with killer-cell immunoglobulin-like receptors (KIR) in a genetically diverse population of sub-Saharan Africans. PLoS Genet.

[45] Salanti A, Clausen TM, Agerbæk MØ, Al Nakouzi N, Dahlbäck M, Oo HZ, Lee S, Gustavsson T, Rich JR, Hedberg BJ, Mao Y, Barington L, Pereira MA (2015). Targeting Human Cancer by a Glycosaminoglycan Binding Malaria Protein. Cancer Cell.

[46] Simon Davis DA, Parish CR (2013). Heparan sulfate: a ubiquitous glycosaminoglycan with multiple roles in immunity. Front Immunol.

[47] Rabinovich GA, Baum LG, Tinari N, Paganelli R, Natoli C, Liu FT, Iacobelli S (2002). Galectins and their ligands: amplifiers, silencers or tuners of the inflammatory response?. Trends Immunol.

[48] Lowe JB (2001). Glycosylation, immunity, and autoimmunity. Cell.

[49] Daniels MA, Hogquist KA, Jameson SC (2002). Sweet “n” sour: the impact of differential glycosylation on T cell responses. Nat Immunol.

[50] Demetriou M, Granovsky M, Quaggin S, Dennis JW (2001). Negative regulation of T cell activation and autoimmunity by Mgat5 N-glycosylation. Nature.

[51] Gauthier L, Rossi B, Roux F, Termine E, Schiff C (2002). Galectin-1 is a stromal cell ligand of the pre-B cell receptor (BCR) implicated in synapse formation between pre-B and stromal cells and in pre-BCR triggering. Proc Natl Acad Sci U S A.

[52] Nguyen JT, Evans DP, Galvan M, Pace KE, Leitenberg D, Bui TN, Baum LG (2001). CD45 modulates galectin-1-induced T cell death: regulation by expression of core 2 O-glycans. J Immunol Baltim Md 1950.

[53] Nielsen N, Ødum N, Ursø B, Lanier LL, Spee P (2012). Cytotoxicity of CD56(bright) NK cells towards autologous activated CD4+ T cells is mediated through NKG2D, LFA-1 and TRAIL and dampened via CD94/NKG2A. PloS One.

[54] Zamai L, Ahmad M, Bennett IM, Azzoni L, Alnemri ES, Perussia B (1998). Natural killer (NK) cell-mediated cytotoxicity: differential use of TRAIL and Fas ligand by immature and mature primary human NK cells. J Exp Med.

[55] Jacobs R, Hintzen G, Kemper A, Beul K, Kempf S, Behrens G, Sykora KW, Schmidt RE (2001). CD56bright cells differ in their KIR repertoire and cytotoxic features from CD56dim NK cells. Eur J Immunol.

[56] Nagler A, Lanier LL, Cwirla S, Phillips JH (1989). Comparative studies of human FcRIII-positive and negative natural killer cells. J Immunol.

[57] Ellis TM, Fisher RI (1989). Functional heterogeneity of Leu 19”bright”+ and Leu 19”dim”+ lymphokine-activated killer cells. J Immunol.

[58] Aramburu J, Azzoni L, Rao A, Perussia B (1995). Activation and expression of the nuclear factors of activated T cells, NFATp and NFATc, in human natural killer cells: regulation upon CD16 ligand binding. J Exp Med.

[59] Shaw JP, Utz PJ, Durand DB, Toole JJ, Emmel EA, Crabtree GR (1988). Identification of a putative regulator of early T cell activation genes. Science.

[60] Durand DB, Shaw JP, Bush MR, Replogle RE, Belagaje R, Crabtree GR (1988). Characterization of antigen receptor response elements within the interleukin-2 enhancer. Mol Cell Biol.

[61] Jauliac S, López-Rodriguez C, Shaw LM, Brown LF, Rao A, Toker A (2002). The role of NFAT transcription factors in integrin-mediated carcinoma invasion. Nat Cell Biol.

[62] Robbs BK, Cruz ALS, Werneck MBF, Mognol GP, Viola JPB (2008). Dual roles for NFAT transcription factor genes as oncogenes and tumor suppressors. Mol Cell Biol.

[63] Wu H, Peisley A, Graef IA, Crabtree GR (2007). NFAT signaling and the invention of vertebrates. Trends Cell Biol.

[64] Peng SL, Gerth AJ, Ranger AM, Glimcher LH (2001). NFATc1 and NFATc2 together control both T and B cell activation and differentiation. Immunity.

[65] Rothfelder K, Märklin M, Wild J, Dörfel D, Kanz L, Müller MR, Salih HR (2016). Involvement of NFAT Transcription Factors in NK Cell Reactivity. Blood.

[66] Pallmer K, Oxenius A (2016). Recognition and Regulation of T Cells by NK Cells. Front Immunol.

[67] Schuster IS, Wikstrom ME, Brizard G, Coudert JD, Estcourt MJ, Manzur M, O’Reilly LA, Smyth MJ, Trapani JA, Hill GR, Andoniou CE, Degli-Esposti MA (2014). TRAIL+ NK cells control CD4+ T cell responses during chronic viral infection to limit autoimmunity. Immunity.

[68] Zhang B, Yamamura T, Kondo T, Fujiwara M, Tabira T (1997). Regulation of experimental autoimmune encephalomyelitis by natural killer (NK) cells. J Exp Med.

[69] Kemp TS (2005). The Origin and Evolution of Mammals.

[70] Power ML, Schulkin J Evolution of Live Birth in Mammals. Evolution of the Human Placenta.

[71] Scheelings TF (2016). Morbidity and mortality of monotremes admitted to the Australian Wildlife Health Centre, Healesville Sanctuary, Australia, 2000-2014. Aust Vet J.

[72] McOrist S, Smales L (1986). Morbidity and mortality of free-living and captive echidnas, Tachyglossus aculeatus (Shaw), in Australia. J Wildl Dis.

[73] Moran S, Vizoso M, Martinez-Cardús A, Gomez A, Matías-Guiu X, Chiavenna SM, Fernandez AG, Esteller M (2014). Validation of DNA methylation profiling in formalin-fixed paraffin-embedded samples using the Infinium HumanMethylation450 Microarray. Epigenetics.

[74] Sandoval J, Heyn H, Moran S, Serra-Musach J, Pujana MA, Bibikova M, Esteller M (2011). Validation of a DNA methylation microarray for 450,000 CpG sites in the human genome. Epigenetics.

[75] Pan M (2011). P44 Expression of calcium/calcineurin pathway-regulated transcription factor NFATc1 and chromatin-remodelling genes BRG1 and BRM in invasive breast cancer. EJC Supplements.

