# Supplementary material for: Epigenetic changes found in uterine decidual and placental tissues can also be found in the breast cancer microenvironment of the same unique patient: description and potential interpretations
Source: Oncotarget. 2017 Dec 19;9(5):6028–41. doi: 10.18632/oncotarget.23488 (PMC5814192; doi:10.18632/oncotarget.23488)
Supplement: Supplementary file 1 [file oncotarget-09-6028-s001.pdf]

Epigenetic changes found in uterine decidual and placental tissues can also be found in the breast cancer microenvironment of the same unique patient: description and potential interpretations

SUPPLEMENTARY MATERIALS

Supplementary Table 1: CpG_islands: with all of the levels of methylation of CpG islands in the genome for the 6 samples (normal breast, normal lymph node, decidua, normal placenta, breast carcinoma and malignant lymph node). In column "Gene", the description of the gene, or genes, affected by the relevant CpG island. (See Methods for more explanation). See Supplementary_Table_1

Supplementary Table 2: (CpG islands) List of Selected Genes with significant Differential Methylation between "self" (normal breast and normal LN) and "non-self" (breast cancer/ metastatic LN and placenta/decidual tissue). See Supplementary_Table_2
